# Improving the Sensory, Nutritional and Physicochemical Quality of Fresh Meat

**DOI:** 10.3390/foods10092060

**Published:** 2021-09-01

**Authors:** Paulo E. S. Munekata

**Affiliations:** Centro Tecnológico de la Carne de Galicia, Rúa Galicia n° 4, Parque Tecnológico de Galicia, San Cibrao das Viñas, 32900 Ourense, Spain; paulosichetti@ceteca.net

This Special Issue titled “Improving the Sensory, Nutritional and Physicochemical Quality of Fresh Meat” is comprised of six studies that explored different strategies to improve the quality of fresh meat, as well as some aspects related to its further processing.

The increasing demand for high quality meat has pushed the professionals and researchers of the meat production area to face new challenges. Consequently, advances covering different stages of the meat production chain process and factors have been made to increase the knowledge and develop strategies to produce and improve the preservation of high quality meat [1,2,3]. Therefore, studying the effect of production systems, diet composition, carcass management, volatile composition of fresh meat related to sensory properties and further processing are topics of interest in attempts to increase knowledge about meat quality and pave the way for strategic changes in the meat industry.

The meat production chain has many stages, starting with the rearing of animals using different systems and diets with optimized composition to favor the animal development and the production of meat with characteristics that are aligned with consumer preferences [3]. Regarding the influence of the production system, Echegaray et al. [4] evaluated the composition and volatile composition of lamb meat produced in an intensive or extensive system. Significant increases in intramuscular fat and protein content in *longissimus thoracis et lumborum* were obtained from animals reared in an extensive system in comparison to animals produced in an intensive system. The extensive system also led to a bigger accumulation of volatile composition in meat in relation to the intensive system. Interestingly, the authors also discussed the relation of intramuscular fat with the composition and content of lipid-derived volatile fraction, wherein these differences were attributed to the presence of specific compounds in each diet (natural antioxidants, for instance) and lipid fraction composition (unsaturated fatty acids and their susceptibility to oxidation).

Another experiment in the context of animal production was carried out by Shin et al. [5] to characterize the effect of stevioside (bioactive compound naturally found in the leaves of *Stevia rebaudiana*) and organic selenium (a crucial component for the endogenous antioxidant system in humans and animals) on the quality of Hanwoo meat. These authors observed significant improvements in animal performance (especially in weight gain and final weight) by using these supplements. The characteristics of meat obtained from animals with the supplemented diet was also improved due to the increase in protein, moisture, PUFA contents, redness, and oxidative stability, and the simultaneous reduction in total cholesterol content, shear force, and drip loss. No major effects in sensory attributes of fresh meat or microbial growth during storage were reported.

Moving forward in the meat production chain, the adequate processing of carcass is a necessary action in order to obtain high-quality meat [1]. In this sense, the experiment conducted by Bakker et al. [6] reported significant improvements in the tenderness and color of beef due to low-voltage electric stimulation of beef carcasses. Moreover, the authors of this study also reported a non-significant impact in cooking loss between the meat control and electrically stimulated carcasses.

The characteristics of meat, especially the sensory attributes, play a central role when consumers judge the acceptance of sensory attributes and the whole eating experience [7]. One of the main factors in this context is the accumulation of unpleasant volatile compounds. This context was considered in the study carried out by Burgeon et al. [8], who evaluated the formation of volatile compounds associated with the perception of boar taint (strong smell found in uncastrated male pigs associated with the presence of skatole and androstenone) in cooked pork. The authors observed higher concentrations of androstenone as the temperature was increased, which indicated a temperature dependence effect. However, the same effect was not reported for skatole. Additionally, studies with further processing of meat were also included in order to explore healthier [9] and functional [10] meat-based foods.

The advances reported in these studies can be seen as meaningful contributions to the generation of knowledge of fresh meat quality and assist, to some extent, in the strategic development of meat production considering the consumer preferences and market trends. Finally, I would like to thank the authors for their submissions; express my sincere gratitude to the reviewers for their time, effort, and insightful suggestions and comments during the reviewing process; and acknowledge the supportive, thoughtful, and expert assistance of the associated editors of Foods throughout the preparation and management of this Special Issue.
